# Gastric DLBCL clonal evolution as function of patient age

**DOI:** 10.3389/fimmu.2022.957170

**Published:** 2022-08-29

**Authors:** Irina Iosselevitch, Hilla Tabibian-Keissar, Iris Barshack, Ramit Mehr

**Affiliations:** ^1^ The Mina and Everard Goodman Faculty of Life Sciences, Bar-Ilan University, Ramat-Gan, Israel; ^2^ Department of Pathology, Sheba Medical Center, Ramat-Gan, Israel; ^3^ Sackler School of Medicine, Tel-Aviv University, Tel-Aviv, Israel

**Keywords:** aging, antigen receptor repertoire, B lymphocytes, gastric diffuse large B cell lymphoma (DLBCL), high-throughput sequencing (HTS), Immunoglobulin (Ig), lineage trees, somatic hypermutation

## Abstract

Diffuse large B cell lymphoma (DLBCL) is the most common type of NHL, accounting for about 40% of NHL cases, and is one of the most aggressive lymphomas. DLBCL is widespread in individuals aged more than 50 years old, with a maximum incidence in the seventh decade, but it may also occur in younger patients. DLBCL may occur in any immune system tissue, including those around the gastrointestinal tract, and even in the stomach, though gastric DLBCL has yet to be sufficiently investigated. This study aimed to understand changes in gastric Diffuse Large B cell lymphoma (gastric DLBCL) development with age. Immunoglobulin (Ig) heavy chain variable region genes were amplified from sections of nine preserved biopsies, from patients whose age varied between 25 and 89 years, sequenced and analyzed. We show first that identification of the malignant clone based on the biopsies is much less certain than was previously assumed; and second that, contrary to expectations, the repertoire of gastric B cell clones is more diverse among the elderly DLBCL patients than among the young.

## Introduction

The gastrointestinal (GI) tract is the most common site of extra-nodal lymphoma, accounting for 40% of cases. DLBCLs frequently arise at extranodal sites, including the gut, and are composed of a diffuse infiltrate of large transformed blasts. Some of these tumors develop from transformed small B cell NHLs, but most arise *de novo* as a result of various genetic events, including gene rearrangements and mutations. Like other DLBCLs, gastric DLBCL can be divided into subtypes such as germinal center B cell-like DLBCL or activated B cell-like DLBCL. Gastric DLBCLs may also contain a low-grade mucosa-associated lymphoid tissue (MALT) lymphoma in the adjacent mucosa and may feature prominent lymphoepithelial lesions (1). DLBCL is a heterogeneous disease with a highly variable clinical course, which is widespread in individuals aged more than 50 years old, with a maximum incidence in the seventh decade, but it may also occur in younger patients; it is currently treated with a combination of immunotherapy and chemotherapy (2). The rearranged IgV genes and intraclonal heterogeneity in DLBCL cells suggest that the cell of origin, which has a prognostic value, mostly comes either from GC B cells or from post-GC B cells (3, 4). DLBCL lineage tree analysis had shown longer mutational history and higher intraclonal diversification, compared with normal control trees (5), but less than MALT lymphoma clones (6). Gene expression profiling (GEP) cannot be routinely used to sub-classify these tumors. Immunochemistry seems the best opinion due to practical/economic reasons. A helpful panel of markers was chosen that may be used for the purpose, however use of these markers to stratify DLBCL into prognostic groups remains controversial (1). Hence, currently High-throughput sequencing (HTS) of Ig variable region genes may be used to identify the malignant clones.

Aging is associated with impairments in anti-inflammatory processes, T cell generation and education, B lymphopoiesis, B cell responses to novel or previously encountered antigens, and increased autoimmunity. The B cell repertoire of the elderly is less diverse than that of young adults, as shown by spectratyping and HTS (7–12). Studies found effects of aging on the BM Hematopoietic stem cells (HSC) compartment and decreased BM lymphocytic cellularity (13). There are fewer niches favorable for sustained lymphopoiesis (10). Defects in AID induction and CSR were observed in B cells of elderly patients (1). B cell output from the BM and the size of the peripheral compartment are regulated by homeostatic pressures imposed by the long lived B cells accumulating in the periphery with age (14, 15) and physiological adjustments. Affinity maturation within GC was also shown to decrease with age in the GCs of Peyer’s patches in the gut (16). The aim of this study was to understand the effects of aging on gastric DLBCL ontogeny and environment by analyzing B cell repertoires and clonal evolution using immunoglobulin (Ig) gene high-throughput sequencing (HTS) and advanced mutational lineage tree-based mutation and selection analyses of Ig gene HTS data extracted from gastric DLBCL biopsies.

## Materials and methods

### Samples

Nine formalin-fixed paraffin-embedded (FFPE) gastric DLBCL biopsies from patients of different ages were selected from the pathology department archive at the Sheba Medical Center. Tissue biopsies were taken during resection procedures and were used in this study in accordance with institutional Helsinki committee guidelines and approval.

### DNA extraction, amplification and sequencing

Experiments were performed as described (6). Briefly, DNA was extracted from 10 sections of FFPE tissue using Qiagene columns (catalog no. 51304) according to the manufacturer protocol with minor changes. Ig heavy chain variable regions were amplified by semi-nested PCR and sequencing was performed by Dyn Diagnostics using the 454-flex titanium instrument. Sequence data were pre-processed as described (6), including the removal of sequences with insertions/deletions (indels) suspected to be caused by the sequencing (a known problem in 454 sequencing), and of duplicate sequences.

### Ig lineage tree analysis

The germline (GL) V(D)J segments of each sequence were identified by SoDA (http://dulci.org/soda/) (17). Groups of sequences containing the same V-D-J gene segments were grouped into clones by our in-house CloneIdentifier program. After finding the consensus GL sequence, all the sequences in the clone and the consensus GL sequence were aligned, to provide parameters for IgTree^©^ (18), such as the regions (complementary-determining and framework regions, CDRs and FWRs, respectively) of each clone. Next, from each alignment of clonally-related sequences, the clonal tree was generated using the IgTree^©^ program developed in the Mehr lab, which is specifically tailored for creating and analyzing Ig gene trees, and the trees were measure using MTree^©^ (19). Finally, our Ig-Indel-Identifier program (20) was used to distinguish between legitimate and artifact indels and to discard sequences that contain suspected artifact indels, and the analysis (from the stage of alignment) was repeated, to ensure correct lineage tree creation (6).

### Clonal size distributions

Groups of sequences containing the same V(D)J gene segments were re-aligned (along with their deduced GL sequences obtained by SoDA) in order to identify clonal relationships, using ClustalW (http://www.ebi.ac.uk/clustalw). Our ClonalSizeDistributionAll.jar program was used to combine the clonal size distributions into one table for every sample. The largest clone of every sample was designated the “dominant clone” (a common term, which we understand to mean one that is larger than all other clones by a significant margin) and assumed to be the malignant clone. The dominant clones were determined by the copy number per unique sequence or – if no copy numbers larger than one were found – by the maximum number of unique sequences. For each biopsy, the dominant clone was compared to other clones obtained from the same biopsy as internal controls, excluding those that may have been parts of the dominant clone

### Mutation identification and lineage tree analysis

In addition to MTree^©^ (19), IgTree^©^ includes additional bioinformatical tools that analyze the mutations in each tree, and identify SHM motifs around mutated positions, in order to examine the mechanism of SHM. Significance of differences between the mutation targeting motifs of dominant and non-dominant clones was examined by the statistical F-test on the ratio of χ² of the two datasets.

## Results

### Samples and sequencing

This study included nine samples of gastric DLBCL, obtained from the archive of the pathology department at the Sheba Medical Center, Israel. Clinical data are summarized in **Table 1**. Ig heavy chain variable region genes were amplified with specific primers and sent to sequencing by 454 Roche. A total of 70416 sequences were analyzed; sequence numbers obtained for each sample and remaining after each stage of processing are given in **Table 2**.

**Table 1:** Gastric DLBCL biopsy information.

Sample	Age	Sex	Clinical background and diagnosis
1	25	F	Overlapping of morphological and phenotypic features between carcinoma and lymphoma. Anaplastic null large cell lymphoma. There were doubts in determining whether it is carcinoma or lymphoma, finally lymphoma was confirmed.
2	34	M	Partial gastrectomy – lymphoma, large cell type, and the tumor is limited to the mucosa.
3	47	F	Ulcerated tumor composed of round cells invading the muscular coats and reaching the serosa. The histologic pictures favor a large cell lymphoma.
4	54	M	Fibrous and granulation tissue covered by necrotic tissue and inflammatory exudate. Clusters of mononuclear atypical, large B lymphoid cells positive on immune-histochemical stains for LC and L26. Features consistent with lymphoma.
5	61	F	Stomach localized large cell lymphoma with ulceration and associated severe chronic and acute inflammation, fibrosis and peritonitis.
6	63	M	Stomach distal part – diffuse large B cell lymphoma involving the entire thickness of the wall up to the perigastric adipose tissue. Lymph nodes of the major curvature are involved.
7	75	M	Stomach (partial gastrectomy) large B cell lymphoma with follicular areas involving the mucosa and submucosa. The surface epithelium is ulcerated. Surgical edges are free of tumor.
8	76	F	Malignant lymphoma B cell, large cell. LC-positive. L26-positive. 4KB5-positive. Keratine-negative.
9	89	F	Biopsy from gastric mass smooth muscle infiltrated by a predominantly large B cell lymphoma with numerous mitoses. Proliferating cell nuclear antigen is positive in 80-90% of the tumor cells.

**Table 2:** Numbers of sequences following each stage of cleaning for all samples^1^.

Sample	Initial no. of sequences	No. of sequences discarded in primer search	No. of unique sequences in the remaining data	No. of sequences with identifiable gene segments	Final No. of sequences (without indels)	No. of clones
1	9563	733	1315	1311	1109	29
2	493	12	283	283	231	26
3	8945	5786	774	767	628	43
4	1575	7	275	275	244	3
5	10396	27	1398	1397	1181	52
6	10935	30	1034	1034	898	16
7	3565	55	1996	1974	1602	123
8	8740	25	1217	1217	1060	36
9	16201	65	4057	4033	2697	241

**^1^** Sequence processing was done according to the protocol described in Materials and Methods. The total numbers of sequences, the numbers of sequences that were discarded at each stage of processing (only one sequence was discarded at the stage of length filtering, from sample 7), the numbers of unique sequences in the remaining data, the numbers of sequences with identifiable V(D)J gene segments as defined by SoDA, the final numbers of sequences that do not contain suspect indels and the numbers of clones obtained from each sample are given.

### Dominant clones are difficult to pinpoint in most biopsies

**Figure 1:**
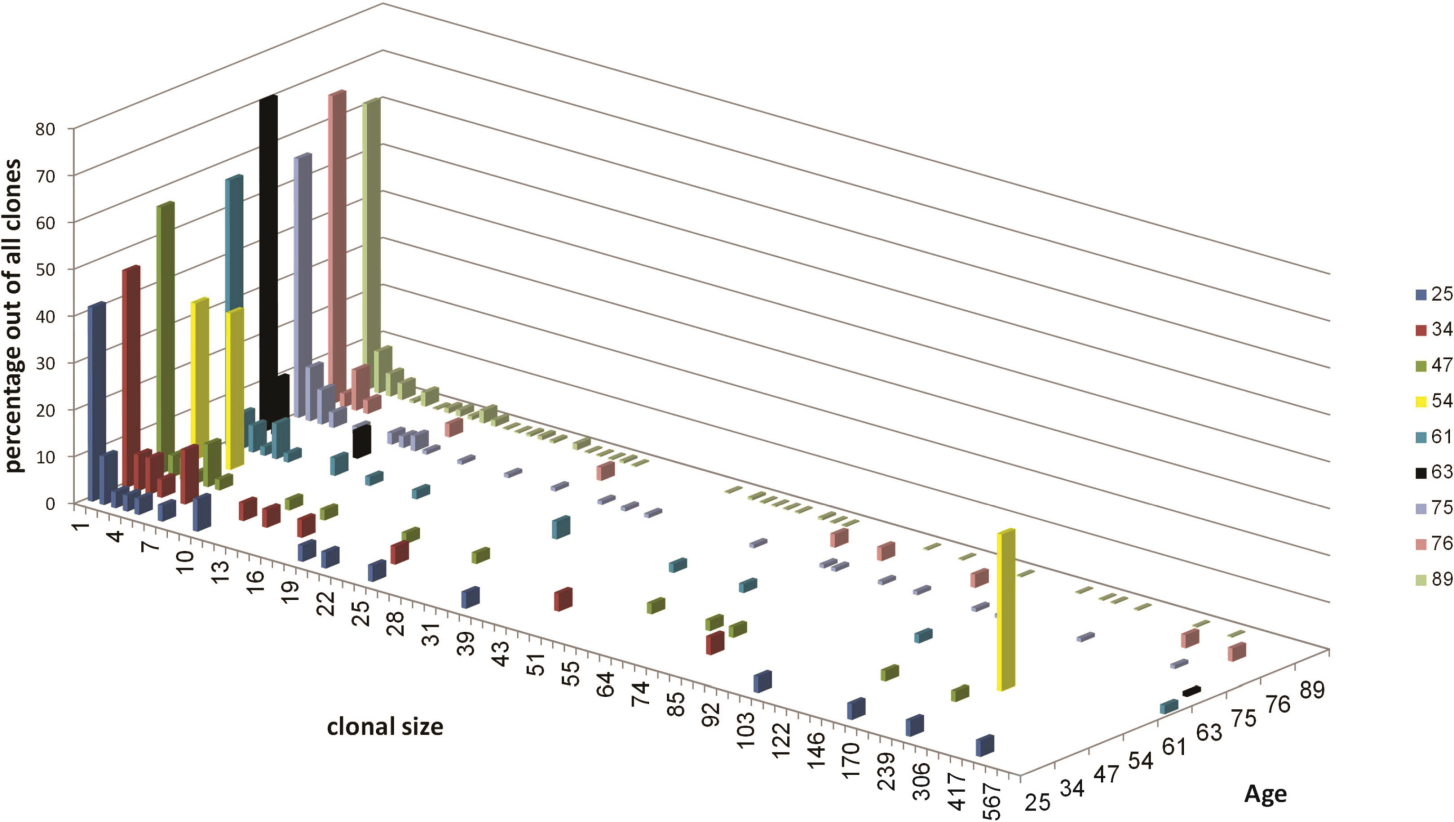
Clonal size distribution for each sample. Different colors represent different samples as indicated in the top right. Clonal size is the number of unique sequences in each clone. The distributions are expressed in terms of the percentages of sequences in each sample that belong to clones of each size, out of the total number of sequences in that sample.

The majority of clones in gastric DLBCL biopsies were small (**Figure 1**). The clonal size distributions show gradual size changes from single unique sequences (clonal size =1, highest column in each sample) to large clones in the majority of samples, except in samples #4 (patient age 54 years) and #6 (patient age 63), each of which contained only one clearly dominant clone. The highest number of clones was obtained from sample #9, followed by sample #7 (ages 89 and 75, respectively). In sample #8 (age 76), the heterogeneity was much lower than that of samples #9 and #7. While all samples contained clones with one unique to several hundred sequences (except in patient #2), the largest clones were found in patients #5 (age 61, 887 sequences) and #6 (age 63, 873 sequences). No correlation was found between maximum clonal size and sample size.

The abundance of large clones in most samples was surprising, as most NHLs have been shown to usually be monoclonal. It is reasonable to expect the malignant clones to take most of the space and to be the largest clones inside tumor tissue, hence we regard only the dominant clone in each patient as the transformed clone (**Table 3**). The non-dominant clones are either clones that contain different V and J segments from those of the dominant clone, or clones that contain the same V or J as the dominant clone, but were grouped separately from the dominant clone because all their intra-clonal distances between sequences were shorter than the distance between those sequences and the dominant clone; the latter clones were excluded from use as controls. Tissue samples are always highly heterogeneous, with the numbers and sizes of clones varying greatly between samples depending on sample size and tissue heterogeneity; this was also the case with our non-dominant clones (**Table 4**). In samples #4 and #6, no non-dominant large clones suitable for comparison were found.

**Table 3:** The dominant clones^1^.

Sample	Clone	copy number per unique sequence	% out of the patient’s sequences	maximum number of unique sequences	% out of clonal unique sequences
1	3-9-01_3-3-01_4-02	164	52.69	438	39.50
	3-11-01_3-10-01_5-02			246	22.18
2	1-8-01_1-26-01_6-02	12	57.66	77	33.33
3	1-8-01_1-26-01_6-02	68	30.54	203	32.32
4	1-69-01_3-22-01_6-02	53	99.18	239	97.95
5	1-69-01_3-22-01_6-02	251	92.79	887	75.11
6	4-4-02_7-27-01_4-02	1335	99.57	873	97.22
7	1-69-04_6-19-01_4-01	68	21.01	417	26.03
8	2-70-10_2-21-01_5-01	157	38.51	282	26.60
	1-18-01_1-26-01_3-02			448	42.26
9	1-2-02_1-1-01_4-02	677	38.69	289	10.72
	5-51-01_1-26-01_4-01			331	12.27

^1^The dominant clones were determined by the copy number per unique sequence or – if no copy numbers larger than one were found – by the maximum number of unique sequences. The percentages of the dominant clone out of all sequences found in each case are also shown.

**Table 4:** Non-dominant clone numbers and average sizes.

Sample	Number of non-dominant clones for comparison	Average clonal size (unique sequences)
1	5	24.667
2	15	6.8
3	25	13.125
5	36	6.222
7	44	10.227
8	17	15.167
9	78	8.579

### The repertoires in gastric tissues of older patients are more diverse compared to those of younger patients

**Figure 2:**
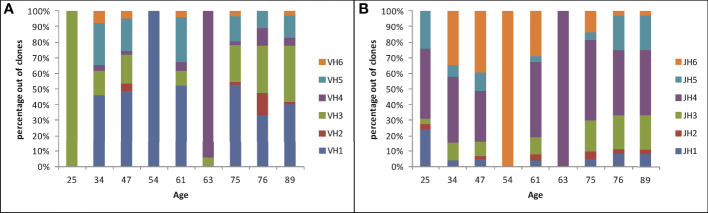
V and J gene family usage in Ig heavy chains in gastric DLBCL vs. age, plotted as the percentages of clones using each gene family in each sample. **(A)** V gene family usage. **(B)** J gene family usage.

The overall clonal repertoire can reveal any potential preference for usage of certain Ig genes inside gastric tissues. We first examined family usage of V and J segments; the D segment is usually too short to reliably determine the identity of the original gene that was used. This revealed a diverse usage of V and J regions in the majority of samples (**Figure 2**), and a large variation in the percent of usage of each gene family among these samples. Sample #4 contained only three clones, which shared the same V and J segments. Sample #1 yielded 29 clones, all of which used of VH3 family genes but with different JH families. Sample #6 included 16 clones, but only one of them used VH3 family and all other clones used VH4 family genes. VH2 and VH6 were rarely used; some biopsies did not contain these families at all. JH4 was the most commonly used J segment, as in normal B cells (12), while JH2 and JH1 were rarely used.

**Figure 3:**
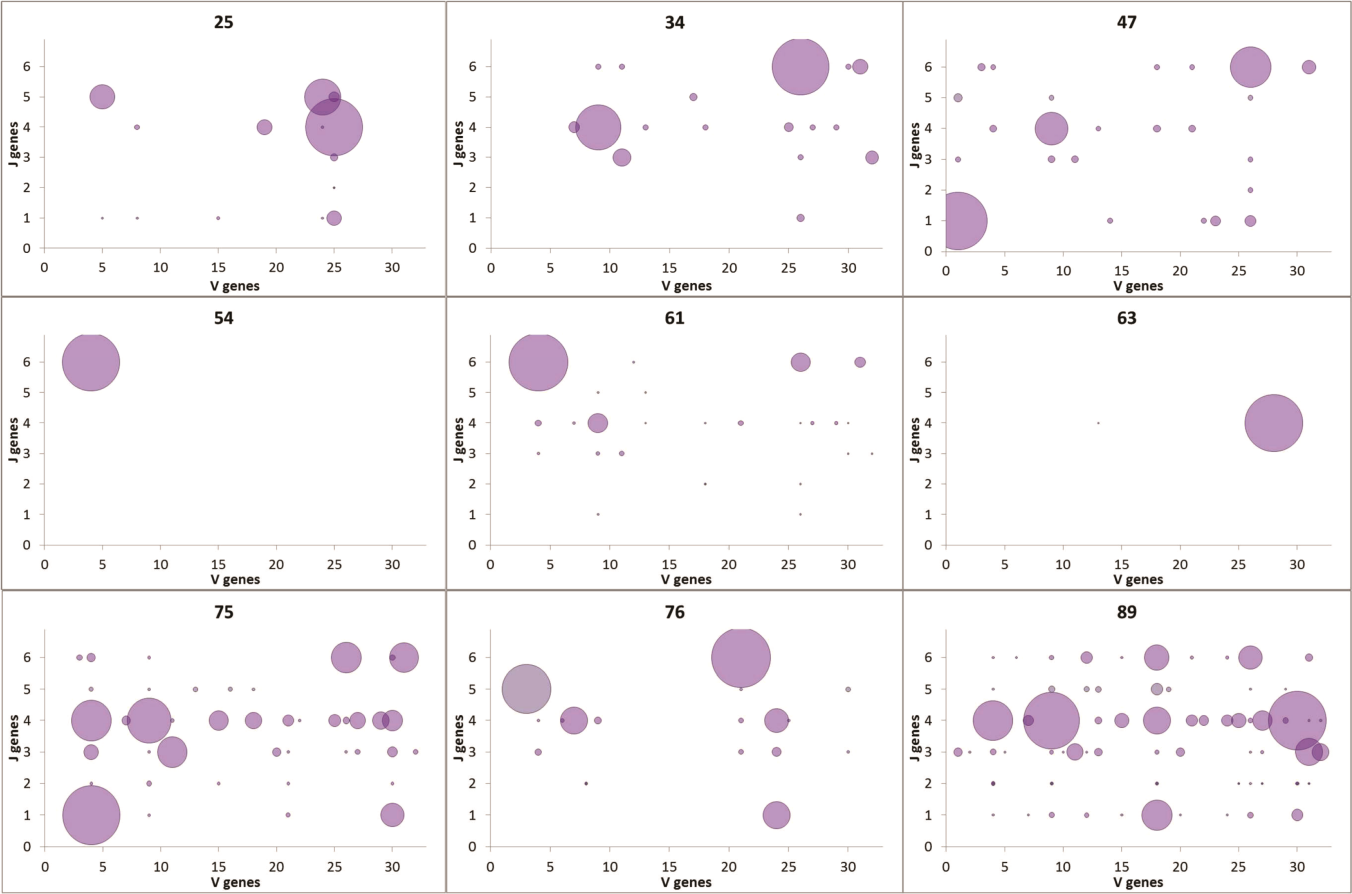
VJ combination usage. V genes are plotted on the x-axis; J genes are plotted on the y-axis. The size of each point represents the number of unique sequences with the particular VJ pairing.

No influence of age on Ig heavy chain gene segment usage was seen. To further understand the observed diversity, we looked at the usage of V-J segment combinations vs. age (**Figure 3**). The gradual distributions of clonal sizes are reflected by the V-J combination usage to some degree, and is especially visible in Samples 7 and 9 of elderly patients (**Figure 3**). Each of the three middle-aged patient samples (#4, #5 and #6) had one large clone and all others were much smaller clones. Almost all samples contained many such small clones, with extremely low numbers of unique sequences, except for #4 and #6, as mentioned above. However, a striking difference between the numbers of V-J segment combinations in samples from young and aged patients was observed, with the elderly having larger diversity, contrary to what occurs in healthy donor repertoires (**Figure 3**).

### Lineage tree analysis suggests higher survival rate and SHM deregulation of dominant clones

**Figure 4:**
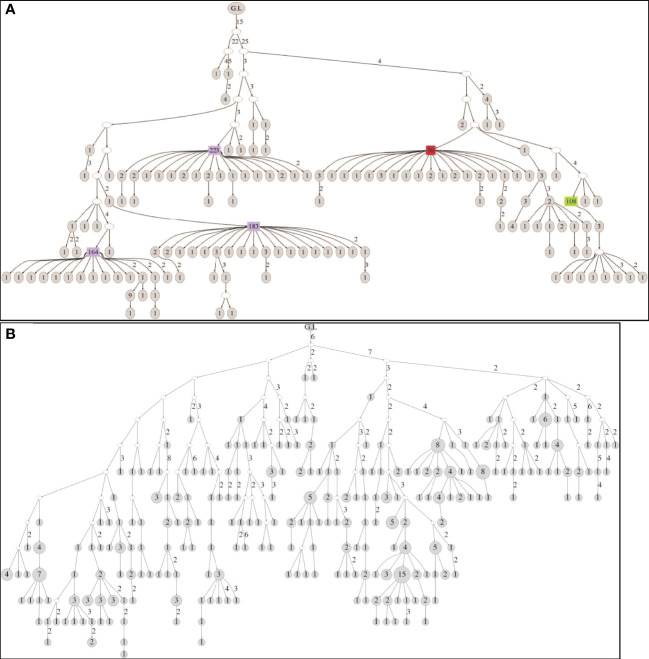
**(A)** Tree structure of a clone obtained from the biopsy of the 25-year-old patient. The clone contains 246 unique sequences and 650 sequences in total. **(B)** Tree structure of a clone obtained from the biopsy of the 89-year-old patient. The clone contains 331 unique sequences and 370 sequences in total. In both figures, G.L signifies the location of the root (germ line). The numbers near edges mean the distance in number of point mutations between the nodes linked. The total number of sequences represented by each node is noted inside the node, with large numbers highlighted in color.

**Figure 5:**
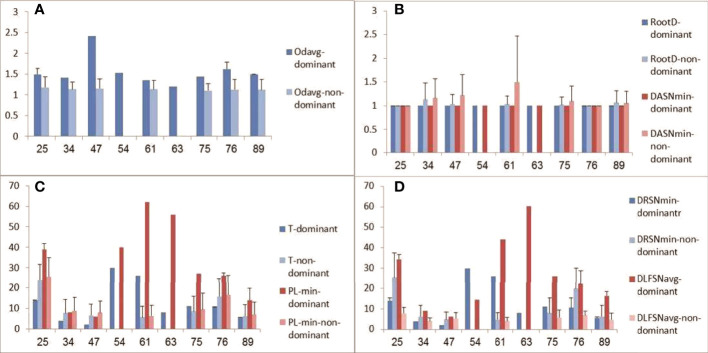
Tree measurements of dominant and non-dominant clones. **(A)** Average outgoing degree (number of children per node); **(B)** root outgoing degree and minimum distance between adjacent split nodes (DASN-min, or fork-to-fork distance); **(C)** trunk length **(T)** and minimum path length (PL-min, from root to a leaf); **(D)** minimum distance from the root to a split node (DRSN-min) and average trunkless path (distance from a leaf to the first split node on the leaf’s path from the root, DLFSN-avg). Patient age is plotted on x-axes, and tree measures (given in numbers of nodes) on y-axes.

Lineage trees were created for all clones, and tree measurements of dominant and non-dominant clones were calculated and compared in every patient. In addition, we looked for changes in tree measurements with age in both dominant and non-dominant clones. The dominant clones showed lineage trees with expanded shapes (**Figure 4**). The degree of branching (average outgoing degree per node, OD-avg) in dominant clones was slightly higher in all cases than that in non-dominant clones (**Figure 5A**), which suggests higher survival rates of mutations in the dominant clones, that is, a higher survival rate of transformed cells. We saw no consistent differences in tree root outgoing degree, which may represent mutation history pre-transformation, nor in the minimum distance between adjacent split nodes, which is an inverse measure of branching (**Figure 5B**). The minimum number of mutations per leaf sequence (minimum path length, PL-min) was also higher in most dominant clones than that in non-dominant clones, which suggests these dominant clones have a longer history of mutations (**Figure 5C**). The same can be concluded from the observation that the average distance from any leaf to the first split node on its path from the root (DLFSN-avg) is higher in dominant clones than in non-dominant ones (**Figure 5D**).

**Figure 6:**
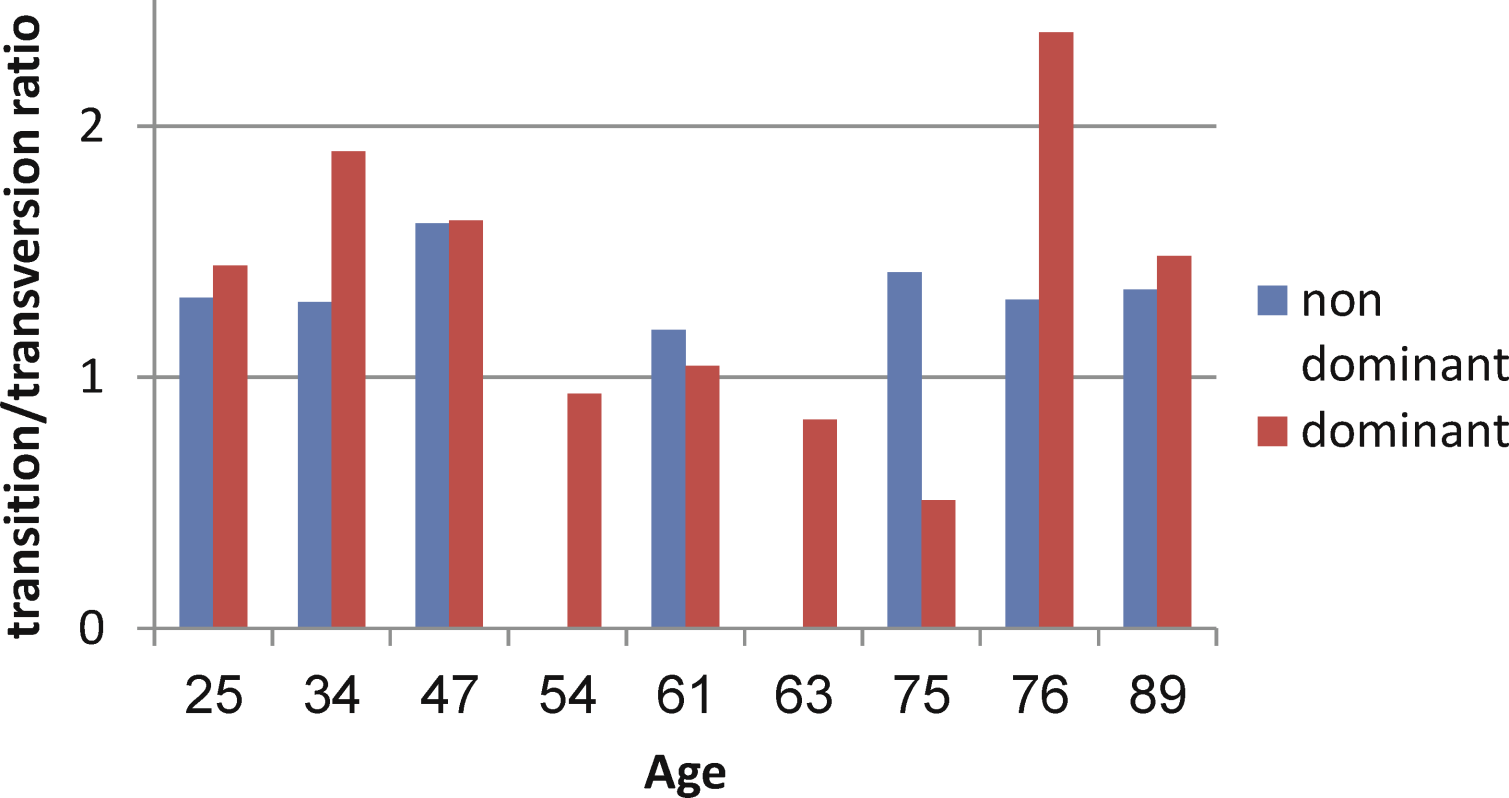
Transition/transversion ratios in dominant and non-dominant clones.

In the next step, we checked whether mutation patterns change with age. In the majority of samples, the transition/transversion ratio was in favor of transition mutations (**Figure 6**). Dominant clones showed a larger range of ratios, while in the non-dominant clones the ratio was always closer to 1. This suggests that SHM mechanisms may be deregulated in some of the malignant clones. No evidence for changes in mutation patterns with age was found.

**Figure 7:**
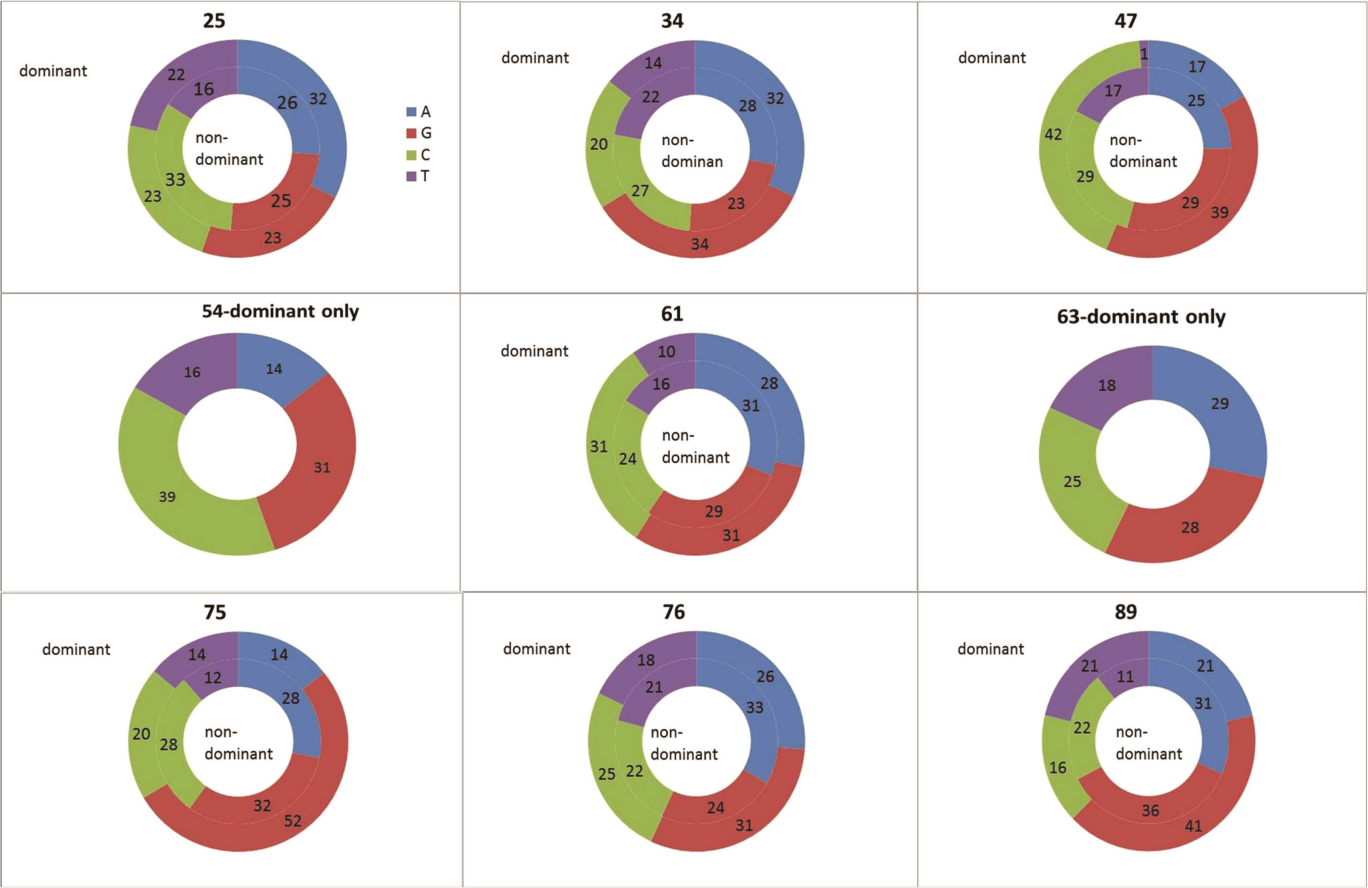
Single nucleotide mutation percentages in dominant and non-dominant clones. The mutation percentages in the dominant clones in each sample are plotted in the outer circles; the mutation percentages in non-dominant clones – in the inner circles. Mutations from Adenine are colored blue; Guanine – red; Cytosine – green; Thymidine – purple.

Single nucleotide mutation patterns were also not uniform (**Figure 7**); the non-dominant clones of all samples and the majority of dominant clones show higher percentages of mutation from purines, except for the dominant clone of the 54-year-old patient. The majority of the samples show higher percentages of mutations from guanine in dominant clones compared to non-dominant clones, except the sample from patient #1, which shows little deviation. This further supports the possibility of deregulation in the mechanisms of SHM in malignant clones.

Targeting of the mutational machinery is biologically important because mutations may modify the hyper-variable regions (CDRs), changing BCR affinity to the Ag. Thus, AID targeting motifs were compared between dominant and non-dominant clones. The dominant clones from all samples were grouped together to create a common motif dataset. The non-dominant clones were grouped as well. The mutation targeting motifs obtained were compared to the reported motifs (21–24). Although the total numbers of point mutations used in this analysis were almost equal between the compared groups, the known mutation targeting motifs clearly appeared (that is, nucleotide over- or under-expression around the mutated nucleotide were statistically significant) in the non-dominant clones, but were not significant in the dominant clones (data not shown). This result again suggests that there may be changes in mutation targeting mechanisms during lymphomagenesis.

## Discussion

This paper makes the following points regarding gastric DLBCL. First, the identification of dominant clones based on preserved lymphoma biopsies is not always straightforward, and even when there is one clearly dominant clone, we cannot be certain the dominant clone is the malignant one based on sequencing Ig gene alone. Second, in contrast to observations in immunologically competent subjects (7, 8, 12, 15), B cell repertoire diversity in gastric lymphoma patients increases rather than decreases with age. Third, SHM and/or selection mechanisms in gastric lymphoma clones may be impaired.

Three samples in this study were shared with a previous study (6). To start with, the data obtained from different sections out of the same sample were analyzed separately in each such case. Contrary to our expectation, the separate analyses did not find overlapping dominant clones. Only one clone was found in two sections from one biopsy; this clone contained a low number of sequences and was probably not malignant. After unification of the data obtained in separate sections, one of the cases had two very large clones (over 200 sequences each); two other samples also contained two large clones each. Different types of B-cell lymphomas and leukemias discussed in literature are classified as monoclonal using different analysis methods, such as Immunofluorescence based on the type of light chain used by B lymphocytes, GeneScan and High Resolution Melting curve analysis (25, 26). In contrast, in the last decade, cases presenting polyclonality were found in age-related EBV-associated DLBCL and chronic lymphocytic leukemia (27–29), casting doubts on the earlier findings. Absence of information can confuse the determination of malignant clones (25). The polyclonality in gastric lymphoma may be explained by the massive amounts of food-related Ag gathering in this tissue. While the stomach, unlike the gut, is not normally surrounded by abundant lymphoid tissues, the immune system does probably deal with the varied types of antigens residing in the stomach, and several reactions can probably happen at the same time, physically close one to another.

As part of dominant clone determination, we checked the location of high copy number sequences in the lineage trees. This was done for the first time in gastric DLBCL, and our finding of non-random locations of the high copy number sequences (**Figure 4**) may be important. Presence of high sequence multiplicity in the node just before the leaves (leaves are the last stage of clonal development before sampling) implies a high number of cells with the exact same Ig heavy chain sequence. In some dominant clones, the maximum number of copies reached several hundreds and even more than a thousand copies of a unique sequence. In the non-dominant clones, such a high copy number was never seen. In addition, high copy numbers were not found in leaves, probably because the cells represented by these sequences have not yet proliferated by the time of sampling. The high numbers of branches coming from the high copy number nodes supports the presence of multiple cells with the same gene, because at least some of the branches probably represent real cells, indicating extensive proliferation and probably malignancy (others may represent PCR errors). Yet, not all the nodes with high copy numbers contained many branches. A loss of information during the preparation process, such as physically cutting the biopsies in the center of the aggregate of the clone’s cells and the dilution of the sample during preparation for sequencing, may also be part of the cause for the paucity of descendants of the leaves in the typical “feather duster”-shaped branches in the dominant clone trees. Further examination of primer bias and PCR errors must be done in future studies, to verify the existence of the high amounts of cells with identical Ig heavy genes and the existence of high numbers of descendants of these cells. The nodes near the roots of the lineage trees also did not contain high copy number sequences. These nodes may represent the pre-malignant transformation stage in clonal development. The cells represented by the nodes near the roots may exist due to their successful antibody having been selected by an Ag. Another explanation for their maintenance in the tissue can be the high motility of the B cells, so that regardless of whether the cells represented by nodes near the root are malignant, their unmutated descendants may be distributed far away from the sampling area.

During aging, the immune system overcomes many changes, many of which influence B lymphocytes (10, 11). Studies have shown a decrease in B cell repertoire diversity and in the output of B lymphopoiesis (10, 12, 15). Damage in processes specific to B cells include defects in AID induction and in CSR (1). No age-related differences in SHM were found in our study. This is consistent with our studies on B cells from immunocompetent subjects (12), where we found that mutation characteristics do not change with age, as opposed to repertoire diversity; and with studies by others (10, 11, 14). Regardless of age, the mechanism of SHM may be deregulated in some of the gastric DLBCL clones. This is expressed by the absence of the main known AID targeting motifs in the dominant clones in our study, while the targeting motifs in non-dominant clones are more similar to the known motifs (data not shown). This assumption is further supported by the variability of the transition/transversion ratios (30, 31) in the dominant clones, contrary to its relative uniformity in non-dominant clones. Because of the high deregulation in the malignant (dominant) clones, any additional influence of aging is probably masked, as opposed to what we saw in normal clones taken from lymph nodes (12, 15).

In a previous gastric DLBCL study, positive antigen selection was found (32). That study was done by a less advanced sequencing method, using only ~20 sequences per case, and using a faulty test that is known to yield many false-positives (33). In contrast, in our study, less selection in the dominant clone can be seen by looking at the higher outgoing degree values in the dominant clones. Further investigation of the issue of clonal selection is needed. In addition, the proportions of replacement and silent mutations should always analyzed after excluding sequences located in the leaves, because they have not yet been subject to selection (34), however this is difficult to do in DLBCL clones which consist of mostly leaves. We are currently examining this issue in wider studies using more advanced sequencing and analysis methods.

The most frequently used VH family in the dominant clones was VH1. The VH6 family was not found in dominant clones in the study. Some of the patients shared the V(D)J rearrangements of the dominant clone: VH(1-69-01)DH(3-22-01)JH(6-02) was shared by two patients, and VH(1-8-01)DH(1-26-01)JH(6-02) was shared by two patients. The VH(1-69) rearrangement was shared by the dominant clones in three of the nine patients. This VH rearrangement was also frequently observed in other lymphomas (35–37), suggesting the potential importance of this gene in gastric lymphomagenesis. The most used JH families in the dominant clones were JH4 and JH6, correlating with other DLBCL studies (38, 39). These genes and combinations should be studied in more detail, as they may be selected by stomach antigens.

The main limitation of this study was the paucity of samples. In spite of the Sheba Medical Center being the largest in Israel and the host of the country’s bio bank, the samples presented here were all we have found at the start of this project. This number of samples, however, could have been enough to detect strong and statistically significant correlations between properties such as dominant clone size and patient age, had there been any. While we completely agree in principle that a larger number of samples would have a higher statistical power for exploring age-related differences in clonal tree properties, we were discouraged from extending this study by our above-mentioned discovery that, in immunologically healthy subjects, the only detectable age-related changes are at the level of B cell repertoire diversity, but not at the clonal level (12).

In summary, we first show that not all gastric DLBCLs may be monoclonal, and the identification of the malignant clone(s) is wrought with problems, at least if assessed by IgHV genes alone based on 454 sequencing; deeper examination of dominant clones, possibly including the use of deeper sequencing methods such as Illumina, sequencing of both IgV regions and using additional markers, and more advanced analysis methods, will be needed for unambiguous identification of the malignant clone(s). In our current studies on DLBCL, we use combinations of these methods [(40), and manuscripts in preparation]. This type of analysis of gastric DLBCL tissues has never been done prior to this work, and the overlapping repertoires in different sections of the same tissue should be studied more deeply in other lymphomas as well, in order to address the question of how representative these biopsy slices are. We also show that, surprisingly, gastric B cell repertoire diversity in elderly gastric DLBCL patients is – at least in this limited study – larger than that in the young patients; and present evidence for potential deregulation of hypermutation and selection in the dominant clones in these patients.

## Data availability statement

Sequence data are available at the NCBI Sequence Read Archive (SRA), BioProject PRJNA855337 (https://www.ncbi.nlm.nih.gov/bioproject/PRJNA855337)

## Author contributions

II did the experiments and wrote the paper; the work was part of her studies towards an MSc degree in Bar-Ilan University. HT-K guided the experimental work and wrote the paper. IB and RM designed the study, supervised the work and wrote the paper. All authors contributed to the article and approved the submitted version.

## Funding

This study was supported by US-Israel Binational Science Foundation (BSF) grant number 20130432 and Israel Science Foundation grant number 270/09 (to RM).

## Acknowledgments

The authors are indebted to Dr. Meirav Kedmi for critical reading of the manuscript, and to Hadas Neuman for help in manuscript preparation.

## Conflict of interest

The authors declare that the research was conducted in the absence of any commercial or financial relationships that could be construed as a potential conflict of interest.

## Publisher’s note

All claims expressed in this article are solely those of the authors and do not necessarily represent those of their affiliated organizations, or those of the publisher, the editors and the reviewers. Any product that may be evaluated in this article, or claim that may be made by its manufacturer, is not guaranteed or endorsed by the publisher.
